# Development of a Species-Specific qPCR Assay for *Theileria annulata* Detection and Its Molecular Surveillance in Kazakhstan

**DOI:** 10.3390/vetsci13101028

**Published:** 2026-09-29

**Authors:** Asylzhan Myrzakhmet, Sabyrkhan Barmak, Kulyaisan Sultankulova, Aidar Namet, Aiganym Tussipova, Timur Davlyatshin, Dulat Inkarbekov, Oralkhan Tusipkanuly, Berik Khairullin, Bekzhassar Sidikhov, Fyodor Vasilevich, Mukhit Orynbayev

**Affiliations:** 1 Scientific Research and Production Center “MVA GROUP” LLP, 1124, Accounting Quarter 096, Koksay Village, Karasay District, Almaty Region 040921, Kazakhstan; a_00_00@mail.ru (A.M.); sultankul70@mail.ru (K.S.); ainamet@mail.ru (A.N.); aiganym.t24@gmail.com (A.T.); avegadti@mail.ru (T.D.); dulat.in@mail.ru (D.I.); oralkan85@mail.ru (O.T.); khirullin@mail.ru (B.K.); omb65@mail.ru (M.O.); 2 Research Institute for Biological Safety Problems, JSC “QazBioPharm” of the Ministry of Health of the Republic of Kazakhstan, Gvardeysky, Kordai District, Zhambyl Region 080409, Kazakhstan; 3 Institute of Veterinary and Agrotechnology, Zhangir Khan West Kazakhstan Agrarian and Technical University, Uralsk 090009, Kazakhstan; 4 Moscow State Academy of Veterinary Medicine and Biotechnology named after K. I. Skryabin, Moscow 109472, Russia; f-vasilevich@mail.ru

**Keywords:** *Theileria annulata*, theileriosis, qPCR, Kazakhstan, cattle

## Abstract

*Theileria annulata* causes tropical theileriosis in cattle and is endemic in Kazakhstan. Although molecular diagnostic methods for this pathogen exist, data on its molecular surveillance and genetic diversity in Kazakhstan remain limited. This study aimed to develop and experimentally validate a species-specific real-time PCR (qPCR) assay for the detection of *Theileria annulata* and to conduct molecular surveillance and phylogenetic analysis of circulating isolates. A total of 709 cattle blood samples from 10 regions of Kazakhstan were analysed. Species-specific primers and a probe were designed targeting the 18S rRNA gene, and diagnostic performance was evaluated against sequencing as the reference method. *Theileria annulata* infection was confirmed in 110 of 709 samples (15.51%), with positive samples identified in the Turkistan, West Kazakhstan, Kyzylorda, and Zhambyl regions. The developed assay showed high diagnostic sensitivity (99.09%), specificity (99.67%), and diagnostic efficiency (99.58%), with no cross-reactivity with *Theileria orientalis*, *Theileria parva*, *Babesia bovis*, *Babesia bigemina*, or *Anaplasma marginale*. Phylogenetic analysis of partial 28S rRNA sequences confirmed the species identity of the detected isolates and demonstrated their close relationship with previously reported Kazakhstani and Chinese isolates. The developed qPCR assay is suitable for the molecular diagnosis and surveillance of bovine tropical theileriosis in Kazakhstan.

## Simple Summary

*Theileria annulata* is a tiny parasite that infects the blood of cattle and causes a serious illness called tropical theileriosis, which is common in Kazakhstan and many other warm-climate countries. Infected animals can become very sick, produce less milk and meat, and sometimes die, causing real financial harm to farmers. Older laboratory methods for finding this parasite, based on examining blood under a microscope, often miss mild infections. In this study, we designed a new, highly accurate genetic test that directly detects the parasite’s DNA in a blood sample. We used this test to check blood from cattle in ten regions of Kazakhstan and found the parasite in four of them. We also compared the genetic makeup of the parasites we found with parasites previously reported in Kazakhstan and China and found them to be closely related. Our new test detected more true infections than the traditional microscope method. These results give veterinarians and animal-health authorities a faster, more reliable tool to find infected cattle early, track how the disease spreads across the country, and take timely action to protect herds and reduce losses for farmers.

## 1. Introduction

*Theileria annulata* is a tick-borne protozoan parasite responsible for tropical, or Mediterranean, theileriosis in cattle. The pathogen is transmitted by several ixodid tick species of the genus *Hyalomma* [1,2,3]. *Theileria annulata* belongs to the genus *Theileria*, family Theileriidae, order Piroplasmida.

Tropical theileriosis causes substantial economic losses worldwide due to reduced animal productivity and increased mortality. The disease is widely distributed in the Middle East [4,5], Asia [6,7], and North Africa [3,8] and poses a particular threat to highly productive imported cattle [9,10]. In Kazakhstan, theileriosis is mainly reported in the Turkistan, Almaty, and Zhambyl regions [11,12,13].

A key epidemiological feature of *Theileria annulata* infection is the ability of recovered cattle to remain persistently infected for prolonged periods, potentially for life. Such carrier animals can act as reservoirs of infection for vector ticks, thereby contributing to the natural transmission and maintenance of the pathogen [14].

Traditional diagnosis of theileriosis is based on microscopic examination of Giemsa-stained blood smears. However, this method is mainly effective when parasitaemia is high. Therefore, more sensitive diagnostic methods are required for accurate assessment of the epizootic situation and implementation of effective control measures [15]. Serological methods based on TAMS and TASP antigens are used primarily to detect specific antibodies [16].

Recent molecular studies confirm the circulation of *Theileria annulata* in Kazakhstan. In a molecular study of 766 cattle from the Turkistan and Zhambyl regions, the overall prevalence of *Theileria annulata* was 83.0% [12]. Circulation of the pathogen has also been confirmed in ixodid ticks. *Theileria annulata* was detected in 8 of 113 examined ticks (7.1%; 95% CI: 3.6–13.4%) from southern regions of Kazakhstan, including Hyalomma scupense from the Zhambyl region and Rhipicephalus annulatus from the Almaty region [17]. These findings confirm the ongoing circulation of *Theileria annulata* among cattle and vector ticks and highlight the need for further molecular monitoring of the pathogen in various regions of Kazakhstan.

Highly sensitive molecular methods based on amplification of different genetic markers have been developed for the detection of *Theileria* spp. in cattle [18,19]. However, despite Kazakhstan being endemic for bovine theileriosis, information on the prevalence, molecular diversity, and circulation of *Theileria* spp. remains limited. Moreover, the lack of complete nucleotide sequences of *Theileria annulata* from Kazakhstan complicates the selection of conserved regions for the development of species-specific oligonucleotides. Accordingly, sequences of the 18S rRNA gene from *Theileria annulata* isolates available in GenBank from Kazakhstan and geographically adjacent regions, including China, Russia, and other parts of Central Asia, were comparatively analysed during primer and probe design.

The 18S rRNA gene was therefore selected as the molecular target for the development of the species-specific qPCR assay because it contains conserved regions suitable for the design of specific primers and a probe. For molecular surveillance, assessment of genetic diversity, and phylogenetic analysis, sequences of the 28S rRNA gene were used because this marker contains variable domains and has greater phylogenetic informativeness. Although promising for phylogenetic studies, the number of available 28S rRNA sequences of *Theileria annulata* remains limited [20]. At present, GenBank contains only a small number of Chinese isolates collected in 2012 and Kazakhstani isolates obtained in 2022–2024. Therefore, the generation of new sequences from Kazakhstani isolates collected in 2025 is of interest for molecular surveillance, expansion of the reference database, and investigation of the genetic diversity of *Theileria annulata* in the region.

The aim of the present study was to develop and experimentally evaluate a species-specific qPCR assay for the detection of *Theileria annulata* and to conduct molecular surveillance and phylogenetic analysis of circulating isolates.

## 2. Materials and Methods

### 2.1. Study Area and Sample Collection

A total of 709 cattle blood samples obtained from 10 regions of the Republic of Kazakhstan were analysed to evaluate the diagnostic performance of the qPCR assay. Blood samples were collected by state veterinary specialists as part of routine epizootological surveillance and were submitted from regional veterinary laboratories for the diagnosis of theileriosis and subsequent molecular genetic analysis.

The study included animals kept on the surveyed farms (regions) and meeting the established inclusion criteria. To assess the actual percentage of infected livestock, animals were selected using simple random sampling. The study included animals regardless of sex, age, and clinical condition, which ensured the representativeness of the sample and excluded preferential selection of animals with clinical signs of disease. After inclusion in the sample, the age, sex, clinical condition, place of keeping, and date of biological material collection were recorded for each animal. Blood samples were collected from cattle between 1 August and 1 December 2025. After collection, samples were labelled and transported to the laboratory in insulated containers with cold packs at +2 to +8 °C. Before molecular testing, blood samples were stored at +4 °C for no longer than 72 h. When longer storage was required, samples were frozen at −20 °C or at −80 °C for long-term storage. Repeated freeze–thaw cycles were avoided. Collection, storage, and transport of biological material were performed in accordance with WOAH recommendations [21,22]. Information on sampling regions, cattle breeds, and sample numbers is presented in Table 1.

### 2.2. DNA Extraction from Blood Samples

DNA was extracted from blood samples positive for *Theileria annulata*; DNA of heterologous haemoparasites was used to evaluate the analytical specificity of the developed qPCR assay. DNA from *Theileria orientalis*, *Theileria parva*, *Babesia bovis*, *Babesia bigemina*, and *Anaplasma marginale* was used for specificity testing. *Theileria annulata* DNA served as the positive control. DNA from clinically healthy cattle blood and deionized water were used as negative controls.

Genomic DNA was extracted from 200 μL of whole blood using the DNeasy Blood & Tissue Kit (Qiagen, Hilden, Germany) according to the manufacturer’s instructions. Nucleic acid concentration was determined using a NanoDrop 2000 spectrophotometer (Thermo Fisher Scientific, Waltham, MA, USA). Purity was assessed using the A260/A280 absorbance ratio. Aliquots of extracted DNA were stored at −20 °C until further use.

### 2.3. 18S rRNA Gene-Based Primer/Probe Design and qPCR Conditions

The 18S rRNA gene of *Theileria annulata*, containing conserved regions suitable for highly specific molecular diagnosis, was selected as the molecular target. Because complete 18S rRNA sequences of *Theileria annulata* from Kazakhstan were unavailable in GenBank, sequences from geographically related regions, including China, Russia, and other parts of Central Asia, were used to identify conserved regions. The performance of the developed assay was subsequently confirmed using Kazakhstani *Theileria annulata* isolates.

Based on sequence alignment, conserved regions were selected to design species-specific oligonucleotides. The following oligonucleotides were developed: forward primer Tan F, 5′-TAATTCCGTTAACGAACGAGAC-3′; reverse primer Tan R, 5′-TTCCTTGCGATTTATAACCG-3′; and probe TanProbe, 5′-FAM-AAGTTTCTACTGTCCCGTTAT-BHQ1-3′.

Amplification was performed using Taq DNA polymerase (Evrogen, Moscow, Russia). The total reaction volume was 25 μL and contained 2.5 μL of 10× reaction buffer, 1.25 μL of MgCl_2_ (50 mM; final concentration, 2.5 mM), 0.5 μL of dNTP mixture (2.5 mM each; final concentration, 50 μM each), 1.0 μL each of Tan F and Tan R primers (10 μM; final concentration, 400 nM each), 1.0 μL of TanProbe (5 μM; final concentration, 200 nM), 0.25 μL of Taq DNA polymerase (1.25 U), template DNA, and nuclease-free water to 25 μL. Cycling conditions were 94 °C for 5 min, followed by 45 cycles of 94 °C for 20 s and 54 °C for 20 s. Amplification was performed on a Rotor-Gene Q real-time PCR cycler (Qiagen), and fluorescence was recorded in the FAM (Green) channel. A pJET1.2 plasmid containing a 111 bp fragment of the *Theileria annulata* 18S rRNA gene was used as the positive control, and nuclease-free water was used as the negative control. Results were interpreted according to the threshold cycle (Ct): samples with Ct ≤ 38 were considered positive for *Theileria annulata* DNA, whereas samples with Ct > 38 or no amplification signal were considered negative.

### 2.4. Microscopic Examination

A thin blood smear was prepared from each sample, air-dried, fixed in absolute methanol for 5 min, and stained with Giemsa solution for 30 min. Microscopic examination was performed using a light microscope (Digital Biological Microscope, BIOBASE, Jinan, China) with a ×100 oil-immersion objective to detect intraerythrocytic forms of *Theileria* spp. At least 20 microscopic fields were examined to confirm a negative result. A sample was considered positive when at least one characteristic *Theileria* piroplasm was observed [23].

### 2.5. Validation of the Developed qPCR Assay

#### 2.5.1. Analytical Sensitivity and Specificity

Analytical sensitivity was determined using a series of 10-fold dilutions of pJET1.2 plasmid DNA containing a 111 bp fragment of the *Theileria annulata* 18S rRNA gene. DNA concentrations in the reaction ranged from 10 ng to 10 ag. Each dilution was tested in triplicate. The limit of detection was defined as the lowest concentration of positive-control material at which amplification was detected in all three replicates with Ct ≤ 38.

Analytical specificity was first evaluated in silico using BLAST (NCBI; web-based BLASTn, https://blast.ncbi.nlm.nih.gov/Blast.cgi, accessed on 30 August 2026) by comparing the oligonucleotide sequences with GenBank data. Specificity was then confirmed experimentally using a DNA panel containing the target pathogen *Theileria annulata*, the phylogenetically related species *Theileria orientalis* and *Theileria parva*, and the epidemiologically relevant haemoparasites *Babesia bovis*, *Babesia bigemina*, and *Anaplasma marginale*. Cattle DNA and deionized water were additionally included as negative controls. Specificity was considered confirmed when no amplification was observed in non-target samples.

#### 2.5.2. Diagnostic Sensitivity and Specificity

Diagnostic sensitivity and specificity were evaluated by comparison with Giemsa-stained blood-smear microscopy, the conventional method of laboratory diagnosis of theileriosis, while positive results were additionally confirmed by sequencing of amplified DNA fragments. Of the 709 biological samples included in the study, 110 were classified as positive and 599 as negative for *Theileria annulata* according to the sequencing reference method. Positive samples originated from the West Kazakhstan (*n* = 29), Zhambyl (*n* = 20), Turkistan (*n* = 35), and Kyzylorda (*n* = 26) regions; all remaining samples were negative.

#### 2.5.3. Repeatability and Reproducibility

Repeatability was assessed from three independent replicate measurements of a positive-control sample within the same qPCR run under identical conditions. Mean Ct, standard deviation (SD), and coefficient of variation (CV, %) were calculated. Reproducibility was evaluated in three independent qPCR runs using the positive-control sample, and mean Ct, SD, and CV were calculated to assess inter-assay variability [24].

### 2.6. Statistical Analysis

Samples positive for *Theileria annulata* by sequencing were classified as true positives (TP), whereas sequencing-negative samples were classified as true negatives (TN). Samples positive by qPCR but negative by sequencing were classified as false positives (FP), and samples negative by qPCR but positive by sequencing were classified as false negatives (FN).

The following performance measures were calculated: sensitivity (SN) = TP/(TP + FN); specificity (SP) = TN/(TN + FP); positive predictive value (PPV) = TP/(TP + FP); negative predictive value (NPV) = TN/(TN + FN); and diagnostic efficiency (DE) = (TP + TN)/(TP + FP + FN + TN). The 95% confidence intervals (CIs) were calculated using the Clopper–Pearson exact method for diagnostic performance indices and the Wilson score method for prevalence estimates [25].

Statistical differences in the detection rate of *Theileria annulata* among the studied regions were assessed using Pearson’s χ^2^ test. Pairwise comparisons between regions were performed using Fisher’s exact test with Holm correction for multiple comparisons.

### 2.7. 28S rRNA Gene-Based Sequencing and Phylogenetic Analysis

Amplification products were sequenced by the Sanger method using the BigDye Terminator v3.1 Cycle Sequencing Kit (Applied Biosystems, Foster City, CA, USA) according to the manufacturer’s instructions. Reaction products were separated on a 3730xl DNA Analyzer (Applied Biosystems, Foster City, CA, USA) and purified using the BigDye XTerminator Purification Kit (Applied Biosystems, Foster City, CA, USA). Sequencing was performed using the same forward and reverse primers as those used for amplification. Chromatograms were analysed using SeqMan (DNASTAR Inc., Madison, WI, USA), including assembly of forward and reverse reads, quality control, and trimming of low-quality terminal regions and primer sequences.

The identities and similarities of sequenced isolates were analysed using BLASTn against the NCBI GenBank database and with MEGA version 11.0 [26]. For phylogenetic analysis, available 28S rRNA sequences of *Theileria annulata* were retrieved from GenBank: PZ830182, PZ830183, PZ830184, PZ830185, PZ312048, PZ312049, PZ312050, PZ312053, PX244311, PX244313, PX244316, PX244317, JN696671, JN696675, JN696676, JN696678, and JN391431.

Multiple sequence alignment was performed using the ClustalW algorithm implemented in MEGA 11. A Neighbor-Joining (NJ) phylogenetic tree was constructed using genetic distances calculated under the Maximum Composite Likelihood model. The reliability of the tree topology was evaluated by bootstrap analysis with 1000 replicates. The *Babesia bovis* sequence JN391431 was used as the outgroup [26].

## 3. Results

### 3.1. Analytical and Diagnostic Performance of the Developed qPCR Assay

The Tan F and Tan R primers and TanProbe were developed to detect *Theileria annulata*. Their performance was confirmed by qPCR, which produced specific amplification of *Theileria annulata* DNA (Figure 1A). Analytical specificity was evaluated using DNA from *Theileria annulata*, *Theileria orientalis*, *Theileria parva*, *Babesia bovis*, *Babesia bigemina*, and *Anaplasma marginale*, together with a negative control. Amplification was observed only in the positive control and samples containing *Theileria annulata* DNA. No specific signal was detected in heterologous haemoparasites or negative controls, indicating the absence of cross-amplification and high analytical specificity (Figure 1B).

Analytical sensitivity was assessed using serial 10-fold dilutions of recombinant pJET1.2 plasmid containing the 111 bp target fragment. Amplification signals were obtained from approximately 3 × 10^9^ to 3 copies of target DNA per reaction. Ct values increased progressively as DNA concentration decreased, from Ct 10.27 at 10 ng to Ct 38.73 at 10 ag. The lowest concentration detected with Ct ≤ 38 was 1 fg (approximately 300 copies per reaction; Ct 36.32), which was defined as the limit of detection. Late amplification signals at 100 ag and 10 ag (approximately 30 and 3 copies; Ct 38.10 and 38.73) were above the positivity cut-off. These results indicate high analytical sensitivity (Figure 1C,D).

The developed qPCR results were compared with blood-smear microscopy, and species identity of positive samples was confirmed by sequencing of an 18S rRNA gene fragment. Among 709 samples, qPCR yielded 109 true-positive, 597 true-negative, 2 false-positive, and 1 false-negative results relative to sequencing. Microscopy yielded 103 true-positive, 597 true-negative, 2 false-positive, and 7 false-negative results (Table 2).

Relative to sequencing, the developed qPCR showed a diagnostic sensitivity of 99.09% (95% CI: 95.04–99.98%), specificity of 99.67% (95% CI: 98.80–99.96%), PPV of 98.20% (95% CI: 93.64–99.78%), NPV of 99.83% (95% CI: 99.07–100.00%), and overall diagnostic efficiency of 99.58% (Table 3). These findings demonstrate the suitability of the developed qPCR assay for the molecular diagnosis of *Theileria annulata*.

For repeatability, Ct values in three parallel determinations were 12.00, 11.93, and 12.22, with a mean of 12.05 ± 0.15 and a CV of 1.26%. For inter-assay reproducibility, Ct values were 12.30, 12.62, and 12.58, with a mean of 12.50 ± 0.17 and a CV of 1.39%. The low CV values indicate high repeatability and reproducibility.

### 3.2. Detection and Geographical Distribution of Theileria annulata

A total of 709 cattle blood samples were analysed by qPCR, and positive results were confirmed by sequencing. *Theileria annulata* infection was identified in 110 samples (15.51%), whereas 599 samples (84.49%) were negative. Positive samples were identified in the West Kazakhstan, Zhambyl, Turkistan, and Kyzylorda regions (Table 4, Figure 2).

A total of 709 samples were tested, of which 110 were positive, corresponding to 15.51% (95% CI: 13.04–18.36%). Positive results were found in 43.75% of the animals examined in the Turkistan region (35/80; 95% CI: 33.41–54.66%), 32.22% in the West Kazakhstan region (29/90; 95% CI: 23.47–42.43%), 26.00% in the Kyzylorda region (26/100; 95% CI: 18.40–35.37%), and 25.00% in the Zhambyl region (20/80; 95% CI: 16.81–35.48%). No positive results were found in the Atyrau, Abai, Almaty, North Kazakhstan, Ulytau, or Karaganda regions. However, the upper limit of the 95% CI in these regions ranged from 4.09% to 10.43%, which does not allow a statistical conclusion of absolute absence of infection in the corresponding populations. The prevalence of theileriosis differed statistically significantly among the studied regions of Kazakhstan (χ^2^ = 147.63; df = 9; *p* < 0.001). In pairwise comparisons using Fisher’s exact test with Holm correction, statistically significant differences were found mainly between regions with detected positive cases and regions with zero prevalence (pHolm < 0.05). No statistically significant differences were found among West Kazakhstan, Zhambyl, Turkistan, and Kyzylorda.

### 3.3. Phylogenetic Analysis of the 28S rRNA Gene

Phylogenetic analysis of partial 28S rRNA gene sequences showed that all *Theileria annulata* isolates analysed in this study clustered within a single clade together with reference *Theileria annulata* sequences deposited in GenBank. The Kazakhstani isolates generated in the present study—PZ830182 (Turkistan 1, cattle, 2025), PZ830183 (Zhambyl 2, cattle, 2025), PZ830184 (WKO 47, cattle, 2025), and PZ830185 (Kyzylorda 53, cattle, 2025)—clustered with previously published Kazakhstani isolates (PZ312048, PZ312049, PZ312050, PZ312053, PX244311, PX244313, PX244316, and PX244317), confirming their assignment to *Theileria annulata* (Figure 3).

The isolates obtained in Kazakhstan in 2025 clustered with previously deposited sequences from Kazakhstan and China (JN696671, JN696675, JN696676, and JN696678), forming a single phylogenetic clade. No pronounced genetic differentiation between isolates from different regions of Kazakhstan and China was observed. The 2025 isolates from the Zhambyl, Kyzylorda, Turkistan, and West Kazakhstan regions showed a high degree of similarity to previously published Kazakhstani isolates from 2022 to 2024 and to Chinese sequences, indicating high conservation of the analysed 28S rRNA fragment.

All *Theileria annulata* sequences were clearly separated from the *Babesia bovis* outgroup, supporting the taxonomic resolution of the analysed marker. Internal tree nodes were supported by maximum bootstrap values (100%), indicating high reliability of the resulting topology and robustness of the *Theileria annulata* clade.

The phylogenetic analysis did not reveal distinct geographically structured clusters among the Kazakhstani and Chinese isolates. This absence of strong geographic clustering suggests high conservation of the analysed 28S rRNA fragment and supports its usefulness for species identification and molecular surveillance, while limiting its ability to resolve intraspecific population differentiation.

## 4. Discussion

Kazakhstan is among the countries affected by bovine theileriosis, partly because its natural and climatic conditions are favourable for the circulation of ixodid ticks, the principal vectors of *Theileria annulata* [12,13,27]. Kazakhstan borders China, Russia, Kyrgyzstan, Uzbekistan, and Turkmenistan, and the movement of agricultural animals may facilitate the spread of pathogens between regions and neighbouring countries [3,28]. Under these conditions, molecular surveillance of *Theileria annulata* is important for timely pathogen detection and improvement of laboratory diagnostics. The present findings confirm the circulation of *Theileria annulata* in the Zhambyl, Turkistan, and Kyzylorda regions of southern Kazakhstan, where theileriosis is endemic, and also in the West Kazakhstan Region.

The 18S rRNA gene was selected as the molecular target for qPCR development because it contains conserved and species-specific regions suitable for primer and probe design. Comparative analysis during assay development was constrained by the limited number of nearly complete 18S rRNA sequences of *Theileria annulata* from Kazakhstan and most Commonwealth of Independent States countries available in GenBank. Only several partial sequences from Kazakhstan (PQ056488–PQ056498) were available, and these did not cover the binding sites of the primers and probe developed in this study [29]. Therefore, conserved regions were selected using available sequences from geographically related areas, including China, Russia, and other countries. Despite this limitation, the assay demonstrated high diagnostic sensitivity and specificity when tested on clinical samples from different regions of Kazakhstan, supporting the validity of the oligonucleotide design strategy.

A recent study [30] developed a panel of species-specific qPCR assays for the detection of *Theileria* spp. based on a variable region of the 18S rRNA gene. The authors demonstrated high diagnostic performance of the method, and the species identity of the detected pathogens was additionally confirmed by Sanger sequencing. The results showed a high level of agreement between qPCR and sequencing data, confirming the value of sequencing as a confirmatory method for assessing the specificity of molecular diagnostics for *Theileria* spp.

In a study conducted in China, a qPCR assay was developed using the TA19140 gene as the molecular target. The developed method was characterized by high sensitivity and specificity and showed no cross-amplification with several closely related hemoparasites. In testing field samples, qPCR provided more effective detection of *Theileria annulata* compared with conventional PCR, and the specificity of the positive amplicons was confirmed by Sanger sequencing [31].

The developed qPCR system had a limit of detection of approximately 300 copies of *Theileria annulata* DNA per reaction, with late amplification signals observed down to 3 copies, confirming its high analytical sensitivity. These characteristics are comparable with those reported for previously published real-time PCR assays for *Theileria annulata*. A quantitative TaqMan PCR targeting the 18S rRNA gene has been reported to show high analytical specificity, no cross-reactions with other *Theileria* and *Babesia* species, a detection limit of 100 target copies, and inter-assay CV values below 3.5% [32]. In a study conducted in China, an 18S rRNA-based TaqMan real-time PCR assay showed no cross-amplification with *Babesia bovis*, *Babesia bigemina*, *Trypanosoma evansi*, and other haemoparasites, with an analytical sensitivity of 10 DNA copies and improved detection compared with conventional PCR [33].

Although the 18S rRNA gene is widely used for molecular diagnosis of *Theileria annulata*, the 28S rRNA gene represents a useful marker for molecular surveillance and phylogenetic studies. Compared with 18S rRNA, the 28S rRNA gene contains a greater number of phylogenetically informative sites and may therefore provide additional information on genetic relationships among isolates [20]. In the present study, 28S rRNA sequences were used to analyse the phylogenetic position of Kazakhstani *Theileria annulata* isolates. The available GenBank dataset previously consisted mainly of Chinese isolates and a limited number of sequences from Kazakhstan, restricting comparative analysis.

The phylogenetic analysis showed that the isolates analysed in this study formed a single clade with previously deposited Kazakhstani and Chinese *Theileria annulata* sequences despite their geographical separation. No pronounced geographic differentiation was detected, suggesting high conservation of the analysed 28S rRNA fragment. Maximum bootstrap support (100%) at the internal nodes indicated high reliability of the inferred clades, and the strong support of the principal lineage confirmed the phylogenetic assignment of the studied isolates.

Only a partial fragment of the 28S rRNA gene was analysed; therefore, the results reflect the conservation of this particular region and do not exclude greater genetic variability elsewhere in the genome. More detailed investigation of the population structure of *Theileria annulata* would benefit from a multilocus approach and a larger number of isolates from different regions of Kazakhstan. However, the studied fragment of the 28S rRNA gene may have limited resolving power for detecting small genetic differences between isolates of the same species. The absence of clear geographic clustering does not rule out the existence of genetic differences among *Theileria annulata* isolates. The use of additional, more variable markers in future studies will allow a more detailed assessment of the genetic diversity of *Theileria annulata* in Kazakhstan.

Overall, the developed qPCR assay can be recommended for the laboratory diagnosis of *Theileria annulata*, and the newly obtained 28S rRNA sequences expand the available molecular genetic database and improve knowledge of the phylogenetic relationships of Kazakhstani isolates.

## 5. Conclusions

A species-specific qPCR assay for the detection of *Theileria annulata* was developed and experimentally evaluated. The assay demonstrated high sensitivity and specificity and was suitable for laboratory diagnosis. Molecular surveillance confirmed the circulation of *Theileria annulata* in four of the 10 regions investigated in Kazakhstan, while phylogenetic analysis demonstrated a close relationship between the detected isolates and previously published sequences. These findings may contribute to improved laboratory diagnosis and molecular surveillance of bovine theileriosis in Kazakhstan.

## Figures and Tables

**Figure 1:**
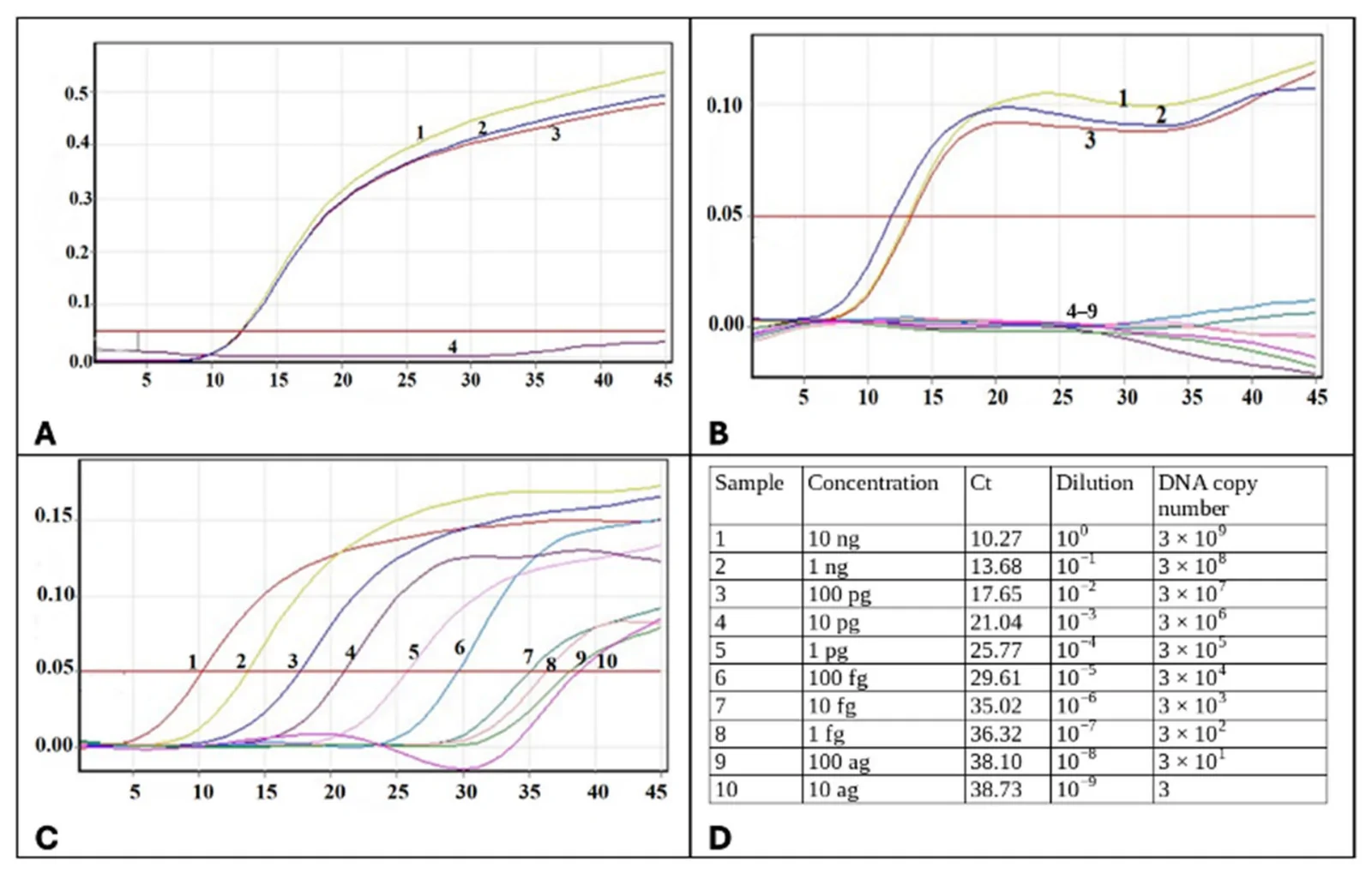
Analytical characteristics of the developed qPCR assay for the detection of *Theileria annulata* DNA. (**A**) qPCR amplification: curve 1, *Theileria annulata* DNA positive control; curves 2–3, positive blood samples; curve 4, negative control. (**B**) Specificity testing: curves 1–2, positive blood samples; curve 3, *Theileria annulata* positive control; curves 4–8, heterologous haemoparasite DNA; curve 9, negative control. (**C**) Serial dilutions from 10 ng to 10 ag. (**D**) Ct values at different DNA concentrations and copy numbers. Amplification curves are identified by numbers; x-axis, PCR cycle number; y-axis, fluorescence (FAM channel); the horizontal line indicates the threshold.

**Figure 2:**
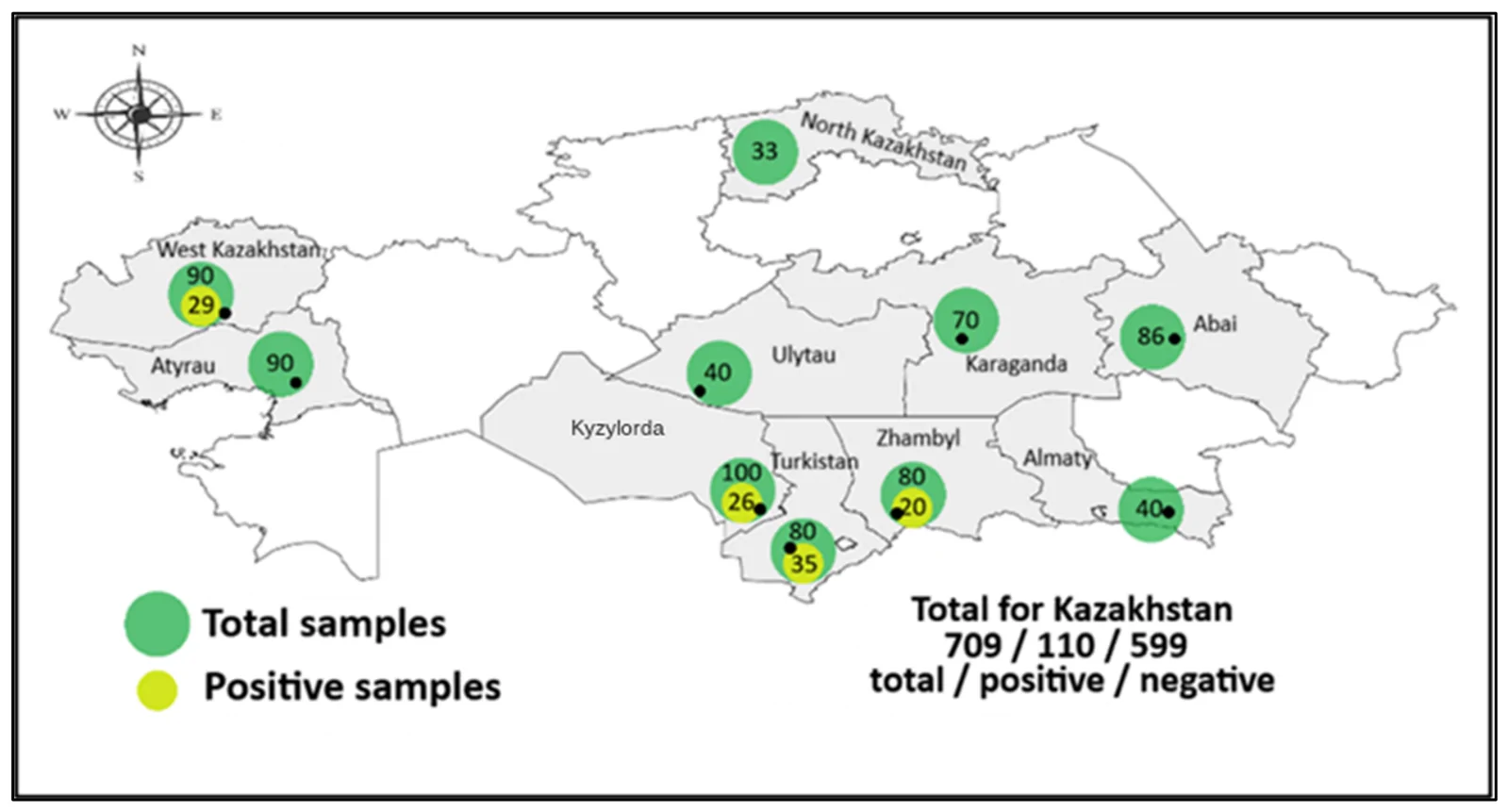
Geographical distribution of cattle blood samples and positive *Theileria annulata* detections in the regions of Kazakhstan. Circles indicate the total number of samples examined and the number of qPCR-positive samples. Cartographic visualization was performed using QGIS 3.40 and OpenStreetMap data (© OpenStreetMap contributors).

**Figure 3:**
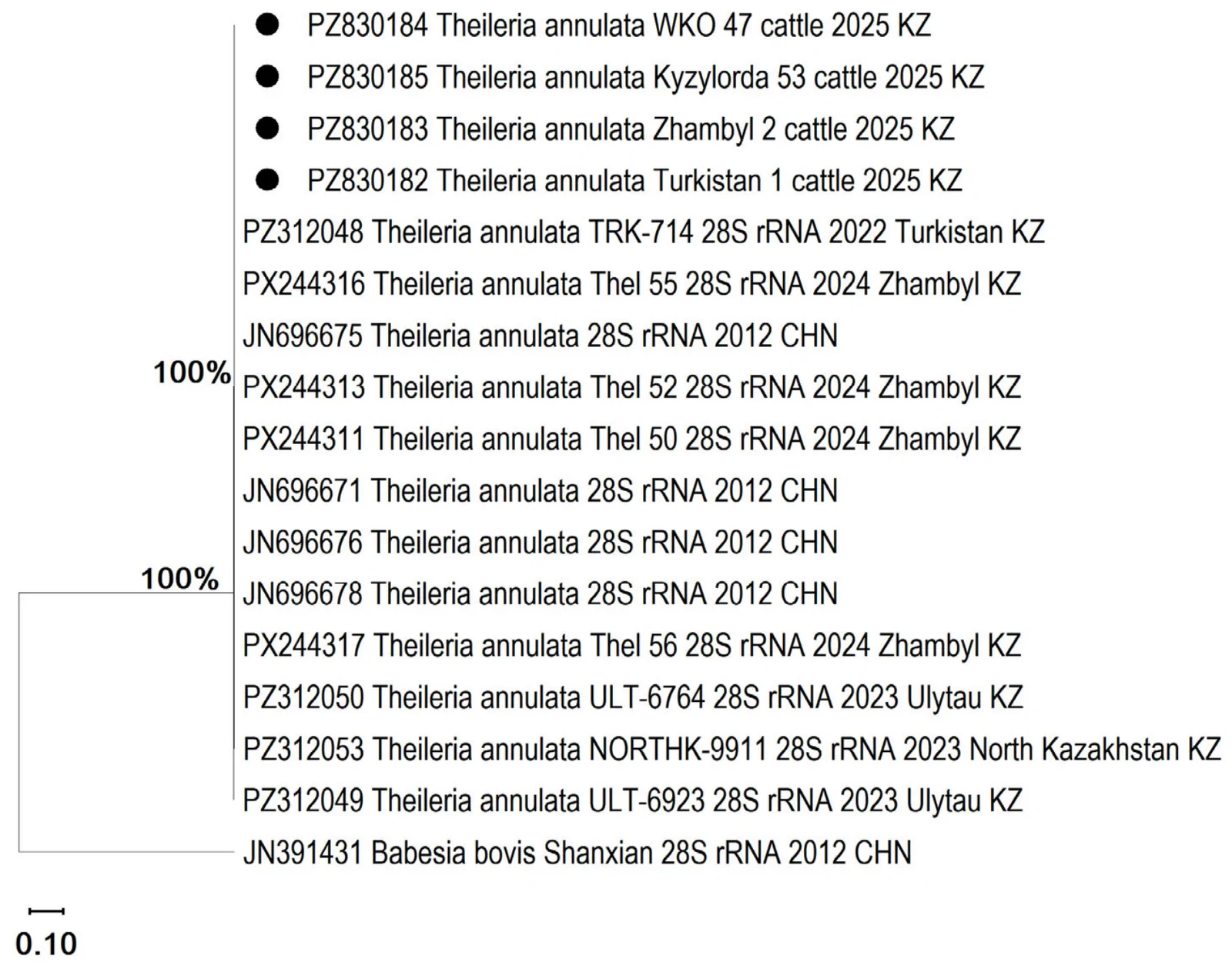
Neighbor-Joining phylogenetic tree based on partial 28S rRNA gene sequences of *Theileria annulata* using genetic distances calculated under the Maximum Composite Likelihood model. Sequences generated in the present study are marked with black circles (●). Numbers at the nodes indicate bootstrap support (1000 replicates). The scale bar indicates the number of nucleotide substitutions per site. *Babesia bovis* (JN391431) was used as the outgroup.

**Table 1:** Biological samples used in the study.

Region	Host/Breed	No. of Samples (*n*)
West Kazakhstan Region	Kazakh White-Headed cattle	90
Zhambyl Region	Black-and-White cattle	80
Turkistan Region	Alatau cattle	80
Kyzylorda Region	Kazakh White-Headed cattle	100
Atyrau Region	Kazakh White-Headed cattle	90
Abai Region	Kazakh White-Headed cattle	86
Almaty Region	Alatau cattle	40
North Kazakhstan Region	Kazakh White-Headed cattle	33
Ulytau Region	Kazakh White-Headed cattle	40
Karaganda Region	Kazakh White-Headed cattle	70
Total		709

**Table 2:** Comparison of qPCR, microscopy, and sequencing results.

Result	qPCR	Microscopy	Sequencing
True positive	109	103	110
False positive	2	2	-
False negative	1	7	-
True negative	597	597	599
Total	709	709	709

**Table 3:** Diagnostic performance of qPCR and microscopy relative to sequencing.

Indicator	Sequencing	qPCR	Microscopy
Total samples	709 (100%)	709 (100%)	709 (100%)
True positives (TP)	110 (15.51%)	109 (15.37%)	103 (14.53%)
True negatives (TN)	599 (84.49%)	597 (84.20%)	597 (84.20%)
False positives (FP)	—	2 (0.28%)	2 (0.28%)
False negatives (FN)	—	1 (0.14%)	7 (0.99%)
Sensitivity, 95% CI	—	99.09% (95.04–99.98)	93.64% (87.33–97.40)
Specificity, 95% CI	—	99.67% (98.80–99.96)	99.67% (98.80–99.96)
PPV, 95% CI	—	98.20% (93.64–99.78)	98.10% (93.29–99.77)
NPV, 95% CI	—	99.83% (99.07–100.00)	98.84% (97.63–99.53)
Diagnostic efficiency	—	99.58%	98.73%

Note: Sequencing was used as the reference method for determining true-positive and true-negative results and for evaluating the diagnostic performance of qPCR and microscopy.

**Table 4:** Geographical distribution of cattle blood samples and positive *Theileria annulata* detections in Kazakhstan.

Region	Total Samples	Positive, n	Positive, %	95% CI
West Kazakhstan	90	29	32.22	23.47–42.43
Zhambyl	80	20	25.00	16.81–35.48
Turkistan	80	35	43.75	33.41–54.66
Kyzylorda	100	26	26.00	18.40–35.37
Atyrau	90	0	0	0.00–4.09
Abai	86	0	0	0.00–4.28
Almaty	40	0	0	0.00–8.76
North Kazakhstan	33	0	0	0.00–10.43
Ulytau	40	0	0	0.00–8.76
Karaganda	70	0	0	0.00–5.20
Total	709	110	15.51	13.04–18.36

## Data Availability

The data presented in this study are openly available in [Theileria_annulata_28S_rRNA] at [https://submit.ncbi.nlm.nih.gov/subs/genbank/SUB16396754/overview], accessed on 30 August 2026, reference number [SUB16396754].
